# Doctor’s recommendations for psychosocial care: Frequency and predictors of recommendations and referrals

**DOI:** 10.1371/journal.pone.0205160

**Published:** 2018-10-04

**Authors:** Jochen Ernst, Hermann Faller, Uwe Koch, Elmar Brähler, Martin Härter, Holger Schulz, Joachim Weis, Norbert Köhler, Andreas Hinz, Anja Mehnert

**Affiliations:** 1 Department of Medical Psychology and Medical Sociology, University Medical Center Leipzig, Leipzig, Germany; 2 Department of Medical Psychology and Psychotherapy, Medical Sociology and Rehabilitation Sciences, and Comprehensive Cancer Center Mainfranken, University of Würzburg, Klinikstrasse 3, Würzburg, Germany; 3 Department and Outpatient Clinic of Medical Psychology, University Medical Center Hamburg-Eppendorf, Martinistrasse 52, Hamburg, Germany; 4 Department of Psychosomatic Medicine and Psychotherapy, University Medical Center Mainz, Untere Zahlbacherstrasse 8, Mainz, Germany; 5 Department of Psychooncology, Clinic for Oncological Rehabilitation, University Medical Center Freiburg, Freiburg, Germany; 6 Clinical Trial Centre, University of Leipzig, Leipzig, Germany; Montpellier Academic Hospital, FRANCE

## Abstract

**Background:**

A significant number of oncological patients are heavily burdened by psychosocial stress. Doctors recommending or referring their patients to psycho-oncologists in the course of routine consultations can positively influence psycho-oncological care. The aim of this study was to analyze the frequency and predictors of such recommendations and to examine the use of these services by patients.

**Methods:**

4,020 cancer patients (mean age 58 years; 51% women) were evaluated in a multicenter, cross-sectional study in Germany. Data was gathered about doctors’ referral practices, patients’ utilization of psycho-oncological care services, and disease-related symptoms. The PHQ-9 depression scale and the GAD-7 anxiety scale were used to measure psychological burden. Descriptive data analysis was conducted on the basis of subgroup comparisons and multivariable analysis was done using binary logistical regression.

**Results:**

21.9% of the respondents reported having been given a recommendation or referral for psycho-oncological care by a doctor within the course of their cancer diagnosis and treatment. This comprises 29.5% of the patients identified by screening as being psychologically burdened. Nearly half of the patients who received a recommendation or referral (49.8%) acted on it. Predictors for seeking out psycho-oncological care included: patient desire (OR = 2.0), previous experience with psycho-oncological care (OR = 1.59), and female gender (OR = 1.57). Multivariable analysis indicated that patients’ level of psychological burden (depression, anxiety) had no effect on whether doctors gave them a recommendation or referral.

**Conclusions:**

Along with examining the degree to which patients are burdened (e.g. using screening instruments), determining whether or not patients would like to receive psycho-oncological care is an important aspect of improving referral practices and, by extension, will allow important progress in the field of psycho-oncological care to be made.

## Introduction

About 8–24% of cancer patients experience depression and 17–19% suffer from anxiety disorders or other significant mental impairments [1–3]. One in every three cancer patients have a clinically relevant mental health disorder (4-week prevalence) [4] and prevalence rates of psychological comorbidity are estimated at 39.4% [5]. Therefore, psychosocial care tailored to the needs of cancer patients, regardless of which phase of illness they are in, constitutes an important part of providing this population with adequate medical treatment. However, only a fraction of cancer patients experiencing psychosocial impairments are receiving psychosocial care or psycho-oncological counseling. To date, the total percentage of cancer patients in either in- or outpatient treatment settings receiving such services is only 11–30% [6–8].

The discrepancy between the need for psycho-oncological help and the actual use of these services can be explained by barriers from the institutions offering the services or from the patients themselves. Findings on this show that approximately 30% of cancer patients, in fact, do want to receive psychosocial help or some comparable form of service and this need has been found to vary greatly based on cancer type or disease stage [9–11]. A study with 1,300 participants found that patients identified as being distressed were most likely to take advantage of psycho-oncological services if they were younger (up to 50 years old) and living alone or separated from a partner [12].

Multiple studies have reported that, even among people who have been identified as being particularly burdened, only about 28%-51% of these patients report wanting or being willing to accept psychosocial help [6, 13–16]. In one study, 46% of participants who reported not wanting help, justified their response by indicating that they could help themselves. Others indicated that they were already receiving help (24%) or that they did not feel strained enough to require services (23%) [15]. Additional barriers, including a lack of information about psychosocial services or challenges related to accessibility (e.g. geographic distance) were not highly endorsed by patients as reasons for not seeking services. This suggests that, from the perspective of the patients, structural deficits in available services did not play a decisive role in their choice not to avail themselves of that support.

The patients who did take advantage of psycho-oncological services were under significantly more strain and had spent a lot more time undergoing cancer treatment or rehabilitation [6, 15]. Taken altogether, these studies suggest that, the presence of distress, a mental disorder, and a patient’s desire to receive psycho-oncological care are important predictors of whether a patient would seek out psychosocial services. As such, oncologists’ referral practices, especially that of giving concrete recommendations, function as an important connection between oncological and psychosocial care. Simply leaving a remark in a patient’s file for the person’s general practitioner, for example, has no effect on whether they receive psychosocial care [17]. One study conducted on 838 cancer patients found that 30.5% of them had wanted their oncologist to give them a referral for psychosocial care, but only 14.8% actually received one (along with a recommendation). The predictors they identified for referrals being made were: patient unemployment, less severe depressive symptoms, reduced functionality, and patient desire for a referral [18].

Taken altogether, the current state of research does not offer consistent answers concerning the influence a patient’s level of psychological burden has on their doctor’s likelihood of referring him/her to psychosocial care. The material below presents an epidemiological study of patients’ reports on doctors’ practices of referring cancer patients to psycho-oncological services.

### Research questions

1. How often do doctors recommend psychosocial support or refer their patients to psycho-oncological services, as reported by patients, and how frequently do patients actually use these services?
2. Which (1) sociodemographic, (2) psychosocial, and (3) disease-related factors predict whether a patient will be given a recommendation or referral by their doctor?

## Methods

The methods of the study are described in detail elsewhere [19]. In this multicenter, epidemiological cross‐sectional study, we recruited cancer patients from acute care hospitals, outpatient facilities, and cancer rehabilitation clinics across 5 study centers in Germany (Freiburg, Hamburg, Heidelberg, Leipzig, Schleswig-Holstein and Würzburg). A total of 4,020 patients were included in this study. The study complied with the Declaration of Helsinki and was approved by the ethics committees of all participating centers: Hamburg: Ref. Nr. 2768; Schleswig-Holstein: Ref. Nr. 61/09; Freiburg: Ref. Nr. 244/07; Heidelberg: Ref. Nr. S-228/2007; 50155039; Würzburg: Ref. Nr. 107/07; Leipzig: Ref. Nr. 200–2007.

### Study participants

Inclusion criteria consisted of being a cancer patient, between the ages of 18–75, with a malignant tumor. Patients across all tumor entities and disease stages were included and were stratified by nationwide incidence of cancer diagnoses. All participants provided written informed consent before taking part in the study.

### Measures

#### Received recommendation or referral for psychosocial support

To assess for the presence of a referral or recommendation, patients were asked: “In the course of having cancer, have you ever received a recommendation or referral from your doctor for psychosocial support?”. Response options for this question included: “Yes, recommendation”, “Yes, referral”, “Yes, both recommendation and referral”, or “No”. If a participant answered with a response beginning with “yes” to the above question they were prompted with a second question asking if they had acted on their recommendation or referral. If participants selected “no” indicating that they had not acted on their recommendation or referral they were asked why and provided with the following response options: “I don’t need any support”, “I don’t know where to look for such support”, “I didn’t know such services exist”, and “other”.

#### Attitude toward psychosocial support

Participants were asked to rate their attitude toward psychosocial support on a scale ranging from 0 (Negative) to 10 (Positive).

#### Need for and experiences with psychosocial support

To determine their experiences with and current need for psychosocial services, participants were asked: “Do you have a need for psychosocial support” and “Did you ever receive any kind of psychosocial support before you had cancer?”. (Response options: “Yes” or “No”).

#### Psychological distress measures

Depressive symptoms were measured using the reliable and valid Patient Health Questionnaire (PHQ) depression module (PHQ‐9; [20]). This 9-item self-report measure requires patients to rate the presence and severity of nine depressive symptoms that correspond with a depressive episode according to the Diagnostic and Statistical Manual of Mental Disorders, Fourth Revision (DSM‐IV), on a scale ranging from 0 (Not at all) to 3 (Nearly every day). Higher values indicate more severe depressive symptoms. A cut‐off of 9 is most suitable for screening for depressive disorders [21].

Symptoms of anxiety were measured using the reliable and valid Generalized Anxiety Disorder Scale, (GAD‐7; [22]). This 7‐item self‐report questionnaire evaluates the presence of symptoms of a generalized anxiety disorder according to DSM‐IV criteria. Higher values indicate more severe symptoms. A cut‐off of 10 is most suitable for screening for at least moderate anxiety symptoms [23].

### Analysis

For the statistical analysis, descriptive calculations were conducted on the questions assessing whether a recommendation or referral had been received and used.

For the multivariable study of predictors, we calculated binary regression analyses whereby sociodemographic characteristics, psychosocial care, and disease/psychological-related variables were used as independent variables. The dependent variable was a recommendation/referral by the physician (yes/no). In the first step we calculated separate models for the predictor groups: (1) sociodemographic, (2) psychosocial, and (3) disease-related factors. Then we calculated a common model with all the predictors. The Odds Ratio (OR) is presented, which shows whether the likelihood of a particular event (recommendation/referral received: yes) occurring is increased (OR>1) or reduced (OR<1). Data analysis was conducted using the statistics program IBM SPSS, Version 24 [24].

## Results

Table 1 provides an overview of the patients who were included in the study. On average, the participants were 58.1 years old and 49% of them were male. The most frequent diagnoses reported (≥17%) were breast cancer, cancers of the digestive organs, and cancers of the male genital organs.

**Table 1:** Sample characteristics.

N = 4,020	No. of Patients (%)
**Age in years**	
Mean (SD)	58.1 (11.32)
Range	18–75
**Gender**	
Male	1,952 (49%)
Female	2,068 (51%)
**Marital status**	
Single	459 (12%)
Married	2,657 (71%)
Divorced	405 (11%)
Widowed	238 (6%)
No response	261
**Education**	
≤10 years	2,363 (64%)
> 10 years	1,361 (36%)
No response	296
**Tumor site**	
Breast	906 (23%)
Digestive organs	790 (20%)
Male genital organs	677 (17%)
Respiratory and intrathoracic organs	359 (9%)
Female genital organs	317 (8%)
Diseases of the blood and blood forming organs	305 (8%)
Urinary tract	221 (5%)
Lip, oral cavity, and pharynx	113 (3%)
Other	332 (9%)
**Setting**	
Inpatient	1,735 (43%)
Outpatient	1,324 (33%)
Rehabilitation	961 (24%)
**Time since diagnosis (month, SD)**	13.85 (28%)
**Purpose of treatment**	
Curative	2,396 (60%)
Palliative	926 (23%)
Unclear	559 (14%)
No response	139 (4%)

**Fig 1:**
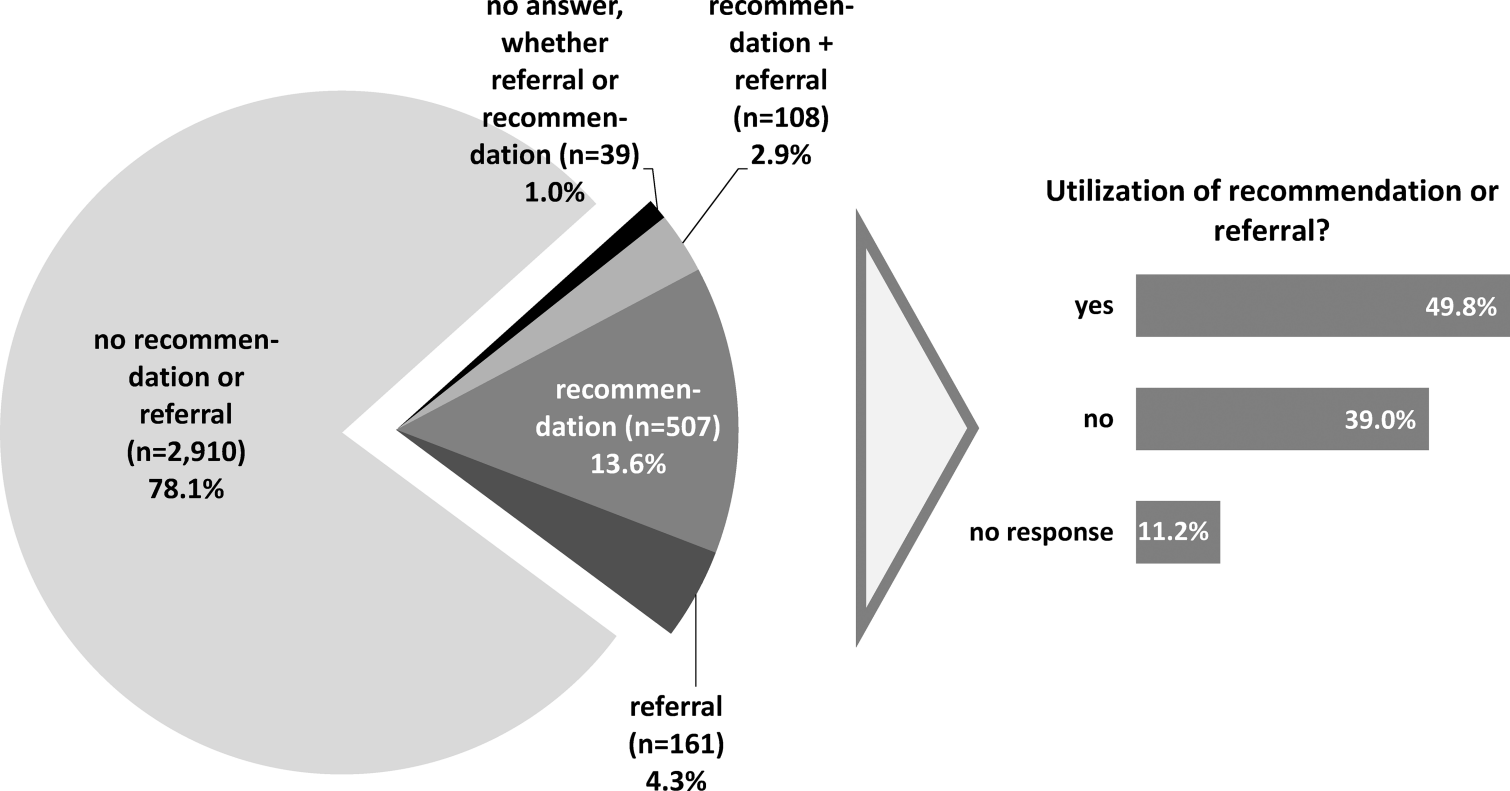
Recommendations or referrals given for psychosocial support services, and utilization of those services (n = 3,725).

507 patients (13.6%) received a recommendation and n = 161 (4.3%) a referral for psychosocial support. 108 patients received both of them (2.9%). 2,910 patients (78.1%) received neither a recommendation nor a referral. The remaining group (n = 295) didn’t respond to the question (Fig 1). Among the participants who indicated having received a recommendation or referral, 49.8% (n = 406) indicated that they had followed the recommendation or acted on the referral. The most frequent reasons given for not acting on their doctor’s recommendation or referral were as follows: (1) They didn’t feel they needed the support (56.0%); (2) They didn’t know where to look for such support (11.9%); and (3) They felt they were already receiving adequate support (6.0%) (multiple responses were possible). Almost one third (29.5%) of the patients who had endorsed symptoms related to psychological distress (i.e., anxiety, depression) had received a recommendation or referral.

A generalized linear model was calculated to assess the impact of age, sex, and psychological burden on a patient’s chances of receiving a referral or recommendation for psychosocial support services (Table 2). The univariate models show the separate effect of the three groups of predictors: (1) sociodemographic variables, (2) psychosocial care-related variables, and (3) disease-related variables. The following factors had a strong effect (OR > 1.5) on recommendation/referral: female gender, higher education, experiences with psychosocial services, and desire for psychosocial support. Weaker effects were found for age and mental health problems (anxiety and depression). The explained variance R^2^ of the models was between 3% and 10%. The resulting final model (4) indicated that the strongest predictors of a patient receiving a recommendation or referral were as follows: (1) patient desire (OR = 2.0); (2) previous experience with psychosocial services (OR = 1.59); and (3) female gender (OR = 1.57). Other significant predictors were: age, education, and positive attitude toward psychosocial support. Degree of psychological burden, time since diagnosis, and level of physical functioning did not play a significant role in influencing whether a patient received a recommendation or referral. The explained variance of the final model (R^2^) was 15.7%.

**Table 2:** Regression: Sociodemographic, psychosocial care-related and disease/psychological-related predictors of doctors of patients’ report of recommending or referring them to psychosocial support services.

**(1) Sociodemographic variables** (n = 3,668)	**OR**	**95% CI**	
*(intercept)*	*1*.*16*	*0*.*75*	*1*.*78*
**Age (decr. per 1 year)**	**1.03**	**1.03**	**1.04**
**Sex: female**	**1.95**	**1.65**	**2.32**
**Education: higher level**	**1.36**	**1.15**	**1.60**
Nagelkerke's R^2^ = 0.084			
**(2) Psychosocial care related variables** (n = 3,556)			
*(intercept)*	*0*.*11*	*0*.*09*	*0*.*13*
**Experience: yes** (previously received support)	**1.64**	**1.30**	**2.07**
**Attitude: positive** (positive attitude)	**1.09**	**1.05**	**1.12**
**Desire: yes** (desire for support)	**2.44**	**2.04**	**2.92**
Nagelkerke's R^2^ = 0.101			
**(3) Disease and psychological related variables** (n = 3,218)			
*(intercept)*	*0*.*21*	*0*.*19*	*0*.*24*
**Time since current diagnosis (per 1 month)**	**1.01**	**1.00**	**1.01**
Karnofsky Performance Status < 80	1.11	0.87	1.40
PHQ-9 ≥ 9	**1.36**	**1.11**	**1.67**
GAD-7 ≥ 10	**1.49**	**1.03**	**2.12**
PHQ-9 ≥ 9 and GAD-7 ≥ 10	1.13	0.72	1.77
Nagelkerke's R^2^ = 0.033			
**(4) Total (all variables)** (n = 3,058)			
*(intercept)*	*0*.*56*	*0*.*32*	*0*.*98*
**Age (decr. per 1 year)**	**1.03**	**1.02**	**1.04**
**Sex: female**	**1.57**	**1.30**	**1.91**
**Education: higher level**	**1.25**	**1.04**	**1.51**
**Experience: yes**	**1.59**	**1.23**	**2.05**
**Attitude: positive**	**1.04**	**1.00**	**1.08**
**Desire: yes**	**2.00**	**1.62**	**2.47**
Time since current diagnosis (per 1 month)	1.01	1.00	1.01
Karnofsky Performance Status < 80	1.19	0.92	1.53
PHQ-9 ≥ 9	1.00	0.80	1.25
GAD-7 ≥ 10	1.08	0.73	1.59
PHQ-9 ≥ 9 and GAD-7 ≥ 10	1.23	0.76	2.01
Nagelkerke's R^2^ = 0.157			

Note: significant predictors are presented in bold.

## Discussion

This study examined the frequency of and factors influencing whether a cancer patient received a recommendation or referral for psychosocial support services from their doctors during the course of their cancer diagnosis and treatment. According to their self-reports, 13.6% of the patients had previously received a recommendation from their doctor for psychosocial support services, and 4.3% received a referral or both (2.9%).

These results are comparable to those reported by other studies [18]. Only about 30% of the patients identified as being psychologically burdened received a recommendation or referral over the course of their medical treatments. This might indicate that doctors are not sufficiently aware of the possible presence or expressions of psychological distress in their patients. One study among breast cancer patients, for example, found that doctors were able to recognize clinically relevant levels of depression in their patients only 24% of the time [25]. In fact, doctors have been found to be accurate in their assessments of how psychologically burdened their patients are only in cases without symptoms (79% accurate assessments in contrast to <33% when mild or severe symptoms were present) [26, 27]. A German study found that the recognition rate of psychological burden in oncological aftercare was particularly poor (<7%) especially when no screening instruments are used [14, 28].

Only about a third of the patients in our study who reported wanting psychosocial support (36%) actually received a recommendation or referral from their doctors. There are various possible explanations for why this rate is rather low. Patients may not explicitly express their desires to their doctors, possibly due to the short length of average consultation times as well as the ratio of time doctors typically spend talking as opposed to listening (as much as ¾ of the time) [29]. It is also possible that doctors did not consider it necessary to further refer their patients because they underestimated the extent of their patients’ psychological burden [25]. Another possibility is that a substantial portion of the patients who were psychologically distressed or had emotional problems may have been hoping to receive psychosocial support from their doctor instead of a psychologist [30, 31].

Almost half of the patients in our study who received a recommendation or referral acted on it. 56% of those who did not act on the recommendation or referral said it was because they felt that they did not need help. This result corresponds with those of other studies reporting low numbers of patients acting on recommendations or referrals despite being psychologically burdened or having a desire to receive psychosocial support [7, 15]. Yet, other studies showed that even in the case of highly burdened patients only about a third of them accept psychosocial support [15] or express a desire to receive such services [13, 32]. The discrepancy between patients’ need for support and their acceptance of services may be influenced by the extent they or other people in their social networks are able to adequately mobilize their own resources [15]. Structural or informational barriers to utilizing psychosocial support services played a relevant role in that study. Correspondingly, only 11.9% of the patients in our study reported not knowing how to find relevant services even after having received a recommendation or referral.

A multivariable analysis of our data revealed that a patient’s desire to receive psychosocial services was the most important predictor of whether their doctor gave them a recommendation or referral (OR = 2.0). Women, participants with higher educational backgrounds, and people who had previous experiences with psychosocial services were also more likely to receive a recommendation or referral. As in other similar studies, these associations were not found to be very strong [32]. Women and highly educated people have more interest in psychosocial care, and they are less reluctant to use these services [33, 34].

The degree to which patients were psychologically burdened (regarding depression and/or anxiety) had no independent impact on their doctors’ recommendation/referral practices. This finding, although interesting, was inconsistent with results reported in previous studies [34–36] and indicates that doctors should not only prioritize identifying psychologically burdened patients in the usual course of consultations but also explicitly ask their patients if they would like to receive psychosocial support [6].

Disease-related factors had no significant influence on obtaining a recommendation/referral. However, in our analyses we only used the variables time since diagnosis and Karnofsky Performance status. In the literature disease-related factors were found to predict the presence of a recommendation/referral. Patients with an early disease stage and patients in current treatment were more likely to receive a recommendation/referral [18, 34]. Furthermore, based on the 15.7% explained variance of the multivariate model, our results suggest that there may be additional predictors influencing whether a doctor recommends or refers their patients to psychosocial care. Further disease-related factors may play a role and should be considered in future research.

The question of which doctors (general practitioners, oncologists, etc.) more frequently recommend psycho-oncological support is also relevant. A high validity of this information could be reached, e.g., when using process data (medical records). Unfortunately, such information was not available in our study.

The limitations of this study include that the data is based on patients’ reports of whether or not they were given a recommendation or referral. Recordings of actual referrals might be a more valid criterion. The results might also be affected by recall bias. Furthermore, when participants were surveyed for our study, they were asked whether or not they had ever received a recommendation/referral in the course of having cancer. By contrast, the extent as to which they were psychologically burdened was measured at the same time that they responded to that question. Thus, we compared recommendations/referrals received up to now with current psychological burden. The major strength of this study is its large sample size, and by extension, the generalizability of its results.

## Implications for clinical practice

Implementing a policy of asking patients about their needs and wishes concerning psychosocial support, as well routinely assessing their levels of psychological burden using economical screening instruments may lead to improvements in referral practices and thereby meaningful progress for psycho-oncological care. This, in turn, may also help to address the issue of doctors frequently underestimating the degree to which their patients are psychologically burdened and in need of support. Referrals should especially be considered in cases where the treatment goals or the patient’s compliance are at risk due to mental health problems.
